# Assignment of Grammatical Gender in Heritage Greek

**DOI:** 10.3389/fpsyg.2021.717449

**Published:** 2021-10-08

**Authors:** Demetris Karayiannis, Maria Kambanaros, Kleanthes K. Grohmann, Artemis Alexiadou

**Affiliations:** ^1^European Master’s in Clinical Linguistics, Faculty of Arts, University of Groningen, Groningen, Netherlands; ^2^Cyprus Acquisition Team (CAT Lab), University of Cyprus, Nicosia, Cyprus; ^3^UniSA Allied Health & Human Performance, University of South Australia, Adelaide, SA, Australia; ^4^Department of English Studies, University of Cyprus, Nicosia, Cyprus; ^5^Institute of English and American Studies, Humboldt-Universität zu Berlin, Berlin, Germany; ^6^Leibniz-Zentrum Allgemeine Sprachwissenschaft (ZAS), Berlin, Germany

**Keywords:** grammatical gender, heritage grammars, Modern Greek, bilingual language acquisition, inflectional class

## Abstract

This study investigates the acquisition of grammatical gender in Heritage Greek as acquired by children (6–8 years of age) and adolescents (15–18 years) growing up in Adelaide, South Australia. The determiner elicitation task from Varlokosta (2005) was employed to assess the role of morphological and semantic cues when it comes to gender assignment for real and novel nouns. Ralli’s (1994) inflectional classes for Greek nouns and Anastasiadi-Symeonidi and Cheila-Markopoulou’s (2003) categories of prototypicality were employed in the analysis of the collected data. The performance of heritage speakers was compared to that of monolingual speakers from Greece (Varlokosta, 2011). The results indicate that–beyond age differences in the two groups–a formal phonological rule guides gender assignment in the production of heritage speakers which departs from initial expectations.

## Introduction

Gender has been argued to be one of the most elusive features of noun phrases (Corbett, 1991). According to Corbett (1991), languages fall into three types when it comes to their gender systems: There are languages with semantic gender systems, languages with predominantly semantic, mixed gender systems, and, finally, languages with formal gender systems. The first two groups use semantic criteria to distinguish between nouns (biological gender), while in the third group of languages, every noun carries gender specification and thus is classified in a particular way. Greek belongs to this group of languages, having a three-gender system, while English has a semantic gender system. Despite its role in nominal classification in this group of languages, as also observed by Picallo (2017), it is not completely straightforward what the role of gender is in a minimalist theory of grammar (Chomsky, 1995 and subsequent work). It is very different from all other types of features, as it does not seem to have a computational import. However, and in spite of the lack of computational import, in languages such as Greek gender seems to be acquired early.

Tsimpli and Hulk (2013) argue that language learners follow two stages in the acquisition of grammatical gender. First, they discover that their language has grammatical gender. Second, they become aware of the fact that gender is a feature to classify nouns in their language and can thus form generalizations and acquire the gender of individual nouns–but importantly also make predictions for novel nouns that they may encounter. While in some languages, such as Dutch, learners have difficulties identifying gender, the acquisition of gender in Greek is effortless, and thus learners acquire the gender system very early on (Tsimpli and Hulk, 2013; Unsworth et al., 2014). For instance, Stephany (1997) reports that gender is acquired by the age of 2:3 (years:months); see also Mastropavlou (2006), who reports a target-like gender system for Greek at the age of 3:6. Neuter is considered to be the default gender for inanimate nouns and in case no agreement can be established (cf. Kazana, 2011; Mastropavlou and Tsimpli, 2011; Anagnostopoulou, 2017; Alexiadou et al., 2021).

Tsimpli and Hulk (2013: 128–129) introduce a further distinction: Next to the learner default (i.e., “the setting adopted by the learner in the earliest stage when input is either unavailable or unanalyzed as yet”), there is the notion of the linguistic default. This notion of a default has also been used to apply to a form which is based on the “elsewhere condition.” From this perspective, it is the less specified value (which applies “elsewhere”) of a particular feature that constitutes the linguistic default. As they argue, neuter is both the learner and the linguistic default in Greek, and this is the reason why its acquisition takes place at a very early age. While Tsimpli and Hulk (2013) did not differentiate between animate and inanimate nouns, Kazana (2011) and Anagnostopoulou (2017) argue that the default is regulated by animacy: In gender resolution contexts involving coordinated nouns, the two groups behave differently; in the case of animate nouns, conjoined singular and plural nouns of the same gender resolve to the gender of the conjuncts. By contrast, conjoined nouns of mixed gender resolve to masculine or feminine if the conjoined nouns denote females. In the case of inanimate nouns, the situation is different: Nouns with the same gender resolve to the gender value of the conjuncts, while inanimate nouns with mixed gender resolve to neuter. This suggests that there is a difference between the learner default and the linguistic default in the case of animates–while the learner default is neuter, the linguistic default is masculine.

This paper reports a pilot study which approaches the notion of the “native speaker” from the heritage language perspective, by focusing on the acquisition of grammatical gender in Heritage Greek in South Australia. The goals of this study were on the one hand to establish whether a determiner elicitation task is a suitable tool for the population in question, and on the other hand to provide novel data on the accuracy of heritage Greek speakers in assigning grammatical gender to real nouns, to investigate whether the lack of semantic information is a significant factor in gender assignment in novel nouns, and to investigate to what extent the same lexical, morphological, and referential principles guide the grammatical gender assignment in the mental grammar of heritage and homeland speakers. After providing the relevant background on heritage linguistics, gender in heritage languages with emphasis on Greek, and the nominal system of Greek, we will present the research (methodology and results), discuss the findings, and briefly conclude.

## Background

### Heritage Linguistics

The role of cross-linguistic influence has been discussed in detail in studies of bilingual language development. Focusing on languages that have gender systems, the main issue has been as to whether or not this would lead to an acceleration or a delay of acquisition. In heritage linguistics, Polinsky (2008) discusses the re-organization of the Russian gender system from a three-gender language to a two-gender language in correlation with the level of proficiency, while Laleko (2019) reports that Russian heritage speakers encounter greater difficulties with underspecified forms, that is, forms that could belong to more than one gender, for which they rely on morpho-phonological criteria.

The discussion of gender in heritage Russian is interesting in the context of Greek, as both languages use a variety of criteria to assign gender: (a) lexical, where certain forms are inherently specified for gender (e.g., *pateras* “father”); (b) morpho-phonological, where certain nouns bear a particular feminine suffix (e.g., *furnar–is–a* “female baker”); and (c) referential, where certain nouns are not specified for gender but receive gender specification via association with a human referent in discourse (see e.g., Alexiadou, 2004, 2017).

Most of the literature on heritage languages focuses on gender agreement. Thus, Polinsky (2018: 206) observes that gender agreement shows effects of vulnerability in heritage speech independently of the gendered vs. un-gendered nature of the language these speakers are dominant in. Montrul et al. (2008: 515) state that “gender agreement appears to be a strong candidate for language loss in a language contact situation.”

There are three studies on heritage Greek which also primarily look at gender agreement. Paspali (2019) tested gender agreement with adult heritage speakers of Greek in Germany, the result being that her speakers were not statistically different from the monolingual controls. Kaltsa et al. (2017) deal with the acquisition of gender assignment and agreement with Greek–English and Greek–German bilinguals. For the gender agreement tasks, their findings suggest that “neuter and masculine are discriminated since the former shows significantly better scores compared to masculine and feminine shows the lowest performance” (p. 24). In addition, they note “that both bilingual groups performed similarly in the gender agreement tasks and were significantly more accurate with neuter than with masculine and feminine suggesting that neuter is treated as the default value, giving rise to fewer errors” (p. 24). Alexiadou et al. (2021) focused on adolescent and adult heritage speakers of Greek in the United States and found that the participants show mismatches in gender agreement, which differ with respect to the agreement target between groups. In particular, noun phrase-internal agreement seems more affected in the adolescent group, while personal pronouns appear equally affected. Their results suggest that heritage speakers resort to neuter gender, which can be attributed to the fact that they have difficulties with establishing agreement chains.

In order to substantiate the use of a default strategy, unlike these previous studies we focus on gender *assignment*, which we investigate in two different age groups.

### The Greek Nominal System

Modern Greek is a highly inflected language. In its nominal system, it syncretically encodes the phi-features of number (singular and plural, henceforth Sg/Pl), case (nominative, accusative, genitive, and vocative, henceforth Nom/Acc/Gen/Voc), and grammatical gender (masculine, feminine, and neuter, henceforth M/F/N). Nouns of native stock and integrated loanwords overwhelmingly fall into eight morphological paradigms, although the number of nouns belonging to each class is not equally distributed. The paradigms are determined by shared morphology across Case and Number (Ralli, 1994, 2002, 2003). These eight inflection classes, henceforth ICs 1–8, are presented in Table 1 (see also Alexiadou and Müller, 2008).

**TABLE 1:** Overview of the Greek inflectional classes according to Ralli (1994).

**IC1 (−*os* M/F)**		**IC2 (−*s* M)**			**IC3 (−Ø F common)**		**IC4 (−Ø F learned)**
o aðelf-os (M)	i isoð-os (F)	o pater-a-s	o kaf-e-s	o xoreft-i-s	i miter-a-Ø	i kor-i-Ø	i poli-Ø
“brother”	“entrace”	“father”	“coffee”	“dancer”	“mother”	“daughter”	“city”
**IC5 (−*o* N)**		**IC6 (−*i* N)**			**IC7 (−*os* N)**		**IC8 (−*ma* N)**
to ner-o “water”		to xtapoð-i “octapus”			to var-os “weight”		to ci-ma “wave”

*The determiner precedes the noun.*

In six out of the eight inflectional classes, the gender-marking suffix in Nom.Sg is realized a consonant (e.g., −*s* for IC2), a vowel (e.g., −*i* for IC6), or a syllable (e.g., −*ma* for IC8). In the case of IC3 and IC4, the presence of a null suffix is proposed in order to elegantly capture the observation of a uniform inflectional paradigm between the feminine nouns with phonological forms ending in/i/and/a/(those vowels being described as parts of the lexical root, or *thematic*), such as *miter-a-Ø*_NOM_ “mother”; *kor-i-Ø*_NOM_ “daughter” and the respective forms *miter-a-s*_GEN_; *kor-i-s*_GEN_ (Ralli, 1994, 2002). The two feminine noun categories are maintained as distinct due to their different plural paradigm in all cases except the genitive: −*es*_*PL*_ for IC3, but −*is*_*PL*_ for IC4. The words in IC4 belong to a more heightened language register but form a productive class. The members of IC8 are deverbal nouns ending in −*ma*. They show imparisyllabic inflected forms with an additional syllable (e.g., *ci.ma*_NOM_; *ci.ma.tos*_GEN_, “wave”). As such they are distinct from some feminine IC4 stems ending in/m/followed by a thematic/a/ (cf. mam-a-Ø, “mom”).

A small number of native words exhibit idiosyncratic declension patterns and do not neatly fit under any IC; moreover, most recent loanwords have no overt inflection (but their phi-features are still valued, as revealed by agreeing determiners and adjectives). The task includes nouns from all eight regular inflectional classes as well as two native lexical items with irregular declension which will subsequently be referred to as “imparisyllabic neuter −*s*,” due to their different syllable count in the genitive singular and all plural forms. These nouns are part of a small non-productive class of neuter nouns bearing the suffix −*s* whose genitive and plural forms are reminiscent of those of IC8 nouns (e.g., *kre.as*_NOM_; *kre.a.tos*_GEN_, “meat”).

The determiner system reflects the same phi-features. They precede and agree with the noun for gender, case, and number. In this study, the nominative singular forms of the definite determiner were elicited: o_M_, i_F_, and to_N_.

The gender system of Greek is described as a primarily formal system, where morphological form, rather than semantic features, predicts the gender value of a given word (Varlokosta and Nerantzini, 2013). This is in opposition to primarily semantic systems, or mixed systems, where the biological sex of the referent is the main predictor of a noun’s grammatical gender, such as in English. The systems can also differ in the extent to which gender is marked across different categories: Greek gender is marked across different nominal and determinative categories, English gender is restricted to the pronominal category.

The role of real-word sex information is elevated under a different categorization system proposed by Anastasiadi-Symeonidi and Cheila-Markopoulou (2003), which relies on the notion of “prototypicality.” According to these authors, certain nouns are prototypically masculine, for example: These are animate, their referent is human, and they bear the morphological ending −*s* (e.g., *pateras* “father”). Other masculine nouns are non-prototypical, and they are inanimate (*çimonas* “winter”). Similarly, prototypical female nouns are animate, their referent is female, and they end in −*a*, −*i*, and −*u*. Finally, inanimate neuter nouns are prototypical and they end in −*o*, −*i*, and −*a*. By contrast, non-prototypical neuter nouns include inanimate nouns ending in −*s* and −*n* and animate nouns (for animals) both inflected (*provato* “sheep”) and uninflected (*koala*) as well as uninflected human nouns (*barman* “barman”).

We note that the suffixes employed by this framework do not line up with those proposed by Ralli (1994), who teases apart gender suffixes that coincide in the citation form but differ during inflection (e.g., *mam–a*_F.NOM_; *mam–as*_F.GEN_ vs. ci-ma_N.NOM_; *cima–tos_N_*_.GEN_). Instead, we adopt Anastasiadi-Symeonidi and Cheila-Markopoulou’s framework in accordance with the design of determiner elicitation task from Varlokosta (2005).

By employing those two systems of categorization, namely Ralli’s exclusively morphological categorization and Anastasiadi-Symeonidi & Cheila-Markopoulou’s system that incorporates semantic as well as morphological cues, we are able to evaluate the contribution of both types of cues separately in the real noun condition.

## Methodology

For the purposes of this pilot study, the accuracy of grammatical gender assignment was measured by eliciting the appropriate definite article form in a determiner–noun context. Varlokosta’s (2005) “Test for Grammatical Gender Assignment to Greek Nouns” was employed, a task which consists of real nouns coming from all ICs as well as novel nouns ending in the seven different possible suffixes for Greek nouns. The participating children were asked to produce the corresponding singular nominative form of the article following a short practice round.

### Participants

A total of 37 young heritage speakers of Greek were recruited in Adelaide, South Australia. The participants were raised and, at the time of testing, resided in Australia. All of them attended English-medium education in Adelaide and as such we consider their dominant language to be English. They were classified in two distinct age groups: 24 children aged 6–8 years (*M* = 8.01, SD = 0.64) and 13 adolescents aged 15–18 years (*M* = 16.77, SD = 1.03). The population in question and the sample of the current pilot study is small and heterogeneous, owing to both the inherent characteristics of the Heritage Greek community of Australia, and the material and time constraints faced by the researchers. No additional factors were considered–such as socio-economic family background, country of birth, age of onset of acquisition for the dominant and heritage languages, formal schooling or literacy in the heritage language, or knowledge of additional languages beyond Greek and English–as priority was given to establishing the appropriateness of the selected task for this novel population.

### Procedure

A single task was administered. The participants were asked to complete the Test for Grammatical Gender Assignment to Greek Nouns (Varlokosta, 2005), which aims to elicit the appropriate form of the nominative singular determiner for 141 items. Of these, 77 items are real nouns (e.g., *ippótis* “knight,” *vrísi* “faucet,” and *város* “weight”) and represent all eight ICs. The remaining 64 items are novel, phonotactically conforming nouns ending in the seven different possible suffixes for Greek nouns (e.g., *péfisma*, *tagherós*, and *oviléða*). Two of the suffixes (−*os* and −*i*) are ambiguous between multiple ICs and possible gender values, while the others are unambiguous. The real and novel nouns constitute two different blocks; the order of items within each block was pseudo-randomized and fixed. The full word-list can be found in Appendix A.

The task was carried out orally, to exclude any facilitatory effect of the Greek morpho-historical spelling, and no vocabulary pre-test was carried out. Each session began with a short training phase consisting of two to four items, depending on the participant’s performance. The aim was to explain to the participants that words in Greek are often accompanied by “a little friend” (the definite determiner). During the introduction, the investigator explained to each participant that “words are often not alone, but instead have a little friend that comes before them”–such as *papús*–*o papús* (“granddad”–“DET_M_ granddad”), *jajá*–*i jajá* (“grandma”–“DET_F_ grandma”), *vivlío*–*to vivlío* (“book”–“DET_N_ book”). Comprehension of the task requirements was tested by using some high-frequency pairs such as *mamá*–*i mamá* (“mum”–“DET_F_ mum”) as training items before the main experiment. One participant from the younger age group who was not able to carry out the task, i.e., to provide a determiner as a response, was excluded from the study.

## Results

### Overview

The collected data were digitized and rated by a native Greek speaker. The two groups showed clear differences in their accuracy when compared against each other and within groups when comparing ICs. Table 2 shows the accuracy of each group per IC in the real noun condition. In Table 3 we report the accuracy per suffix in the novel noun condition and additionally we compare their performance to that of end-state L1 speakers of Greek as reported by Varlokosta (2011) [Note that Varlokosta (2011) only administered the novel nouns sub-task as the study concerned adult monolinguals’ gender assignment performance based solely on the information carried by the noun suffix]. In Appendix C, the Tables C1a–b and C2a–b present individual participant results for both groups in the respective conditions.

**TABLE 2:** Accuracy, mean number, standard deviation, and range of correct responses by child and adolescent Heritage Greek speakers (real nouns).

**Inflectional Category (Items)**	**Children (Accuracy%, *M*, *SD*, Range)**	**Adolescents (Accuracy%, *M*, *SD*, Range)**
**IC1 −*os* M/F (12)**	26.74, 3.21, 1.61, 0–6	48.08, 5.77, 2.28, 3–12
**IC2 −*s* M (12)**	22.57, 2.71, 2.49, 0–12	62.82, 7.54, 4.03, 2–12
**IC3 −Ø F (15)**	33.33, 5.0, 2.54, 2–12	83.08, 12.46, 2.85, 6–15
**IC4 −Ø F (2)**	14.58, 0.29, 0.55, 0–2	88.46, 1.77, 0.44, 1–2
**IC5 −*o* N (11)**	71.21, 7.83, 2.57, 3–11	64.34, 7.08, 3.38, 1–11
**IC6 −*i* N (13)**	74.68, 9.71, 2.63, 5–13	68.05, 8.85, 4.3, 0–13
**IC7 −*os* N (4)**	70.83, 2.83, 1.31, 0–4	42.31, 1.69, 1.18, 0–4
**IC8 −*ma* N (6)**	68.75, 4.13. 1.85, 0–6	51.28, 3.08, 2.53, 0–6
**Imparisyllabic −*s* N (2)**	81.25, 1.63, 0.58, 0–2	42.31, 0.85, 0.9, 0–2

**TABLE 3:** Accuracy, mean number, standard deviation, and range of correct responses by child and adolescent Heritage Greek speakers, compared to the accuracy of typical L1 adult speakers as reported by Varlokosta (2011) (novel nouns).

**Phonological Ending**	**Adults L1 Greek (Varlokosta, 2011)**	**Children Heritage Greek (Accuracy%, *M*, *SD*, Range)**	**Adolescents Heritage Greek (Accuracy%, *M*, *SD*, Range)**
**−*os (12)* as**	100	100, 12, 0, 12–12	100, 1.92, 0.28, 11–12
**M**	85.8 (M)	24.31 (M)	78.71 (M)
**F**	4.3 (F)	16.67 (F)	2.58 (F)
**N**	9.8 (N)	59.03 (N)	18.71 (N)
**−*i* F + N (12)**	99.5 (44.0 F)	83.97, 10.04, 1.81, 6–12 (21.95 F)	92.31, 11.08, 1.12, 9–12 (60.9 F)
**−*is* M (8)**	94.3	20.94, 1.67, 1.99, 0–8	51.92, 4.15, 2.67, 0–8
**−*as* M (8)**	96.0	23.44, 1.88, 1.9, 0–8	59.62, 4.77, 3.17, 0–8
**−*a* F (10)**	91.0	27.62, 2.75, 2.72, 0–10	75.19, 7.46, 2.76, 3–10
**−*o* N (8)**	94.5	61.46, 4.92, 2.48, 1–8	50.96, 4.08, 3.09, 0–8
**−*ma* N (6)**	74.3	50.69, 3.04, 1.99, 0–6	49.35, 2.92, 1.85, 0–6

In order to maintain comparability with the control group’s results as reported by Varlokosta, but also because only the nominative singular form of the novel nouns is presented to the participants, it is not possible to assign the novel nouns to the same ICs that we employ for the real nouns. For example, nouns ending in/os/cannot be assigned to IC1, IC7, or the exceptional imparisyllabic −*s* neuters. Therefore, the responses for all novel nouns with that phonological ending are grouped under a category “−*os* M/F/N.” The feminine IC3 and IC4 and neuter IC6 categories with the phonological ending/i/are also collapsed into one category ambiguous between F and N gender values, as the null suffix that is posited for IC3 and IC4 cannot be assumed to exist in the absence of its consequences on the inflectional paradigm. The neuter suffix −*ma* is treated as unambiguous in relation to feminine nouns of the IC3 category with a null suffix and/a/as their thematic vowel (Varlokosta, 2011; but see the section “Discussion” below for a challenge). Comparison between the real and novel noun conditions is therefore less straightforward by necessity.

The performance per morphological paradigm and prototypicality class (see Table 4) in the real noun condition, and for each suffix in the novel noun condition, will be elaborated on in the following subsections.

**TABLE 4:** Overview of the Greek prototypicality categories according to Anastasiadi-Symeonidi and Cheila-Markopoulou (2003).

	**+Prototypical**	**−Prototypical**
**Masculine**	+animate, male, −*s*	**−**animate, −*s*
**Feminine**	+animate, female, −*a/*−*i/*−*u*	**−**animate, −*a/*−*i*
	**−**animate, abstract, −*a/*−*i*	+animate, professional, −*s*
**Neuter**	**−**animate, −*o/*−*i/*−*a*	**−**animate, −*s*
	+animate, animal and human young, −*o/*−*i*	+animate, non-diminutive animal, −*o/*−*i*

The novel nouns bearing the suffix −os can be plausibly parsed either as IC1 masculine or feminine nouns or as IC7 neuter nouns, therefore any nominative determiner response is coded as accurate and only the failure to provide a determiner is coded as an incorrect response. Similarly, the novel nouns bearing the suffix −i can be plausibly parsed as IC3 or IC4 feminine nouns or as IC6 neuter nouns. A masculine determiner or the failure of providing one is coded as an incorrect response.

In the real noun condition, we observe that the older group (adolescents) performed better than the younger group (children) in ICs 1–4, which included all masculine and feminine nouns, while the children appear more accurate in the neuter ICs 5–8. In all ICs, except for two-item IC4, the child participants’ performance deviates less from the group average compared to the corresponding values for the adolescent group. When examining the overall performance of individual participants, we observe that the younger group’s accuracy across all tasks reached 52% (SD = 0.1), with the worst performing child achieving 30% accuracy and the best, 72%. The adolescent group’s overall accuracy reached 61% (SD = 0.17), with the worst performing adolescent achieving 38% accuracy and the best, 97%.

In the novel noun condition, we observe that for the unambiguous suffixes −*is*, −*as*, −*a*, −*o*, and −*ma*, the adolescents performed better than the children in the masculine and feminine classes, while the children performed better in the neuter −*o* class. The two groups had comparable accuracy in the neuter −*ma* class, but the children performed slightly better in the neuter −*o* class. The performance of individual participants from their respective group averages presents a slightly different picture compared to that for the real nouns. The adolescent group exhibited smaller variance in the phonological endings −*i* and −*ma*. The overall child group accuracy was at 53% (SD = 0.09), with the lowest scoring participant reaching 35% and the highest scoring 73%. The overall accuracy of the adolescent group reached 68% (SD = 0.18), with the lowest scoring participant reaching 43% and the highest scoring reaching 100% accuracy.

While we are not able to compare the two heritage speaker groups to a monolingual homeland speaker control population in the real noun condition, we expect that, in line with the previous studies presented in the section “Introduction,” the homeland Greek speakers will perform near perfectly or at least at the same level as their performance in the novel nouns.

### Accuracy by Inflectional Class of Real Nouns

The test items from IC1 are 12 nouns ending in −*os*, equally split between masculine and feminine. The performance of the two age groups was clearly different, with child participants only being able to produce the correct determiner almost 27% of the time, while the adolescents were successful over 48% of the time. The children erroneously provided a neuter determiner for more than half of the words. The adolescents provided a masculine determiner two thirds of the time but rarely identified words as feminine, instead erroneously producing a neuter determiner one fourth of the time.

There are 12 masculine nouns in IC2 ending in −*s*, of which half are of the −*is* type and the other half of the −*as* type. Most children were unable to correctly identify these nouns as masculine, providing a neuter determiner at more than 61%, and a feminine determiner at over 15%. The adolescents were successful in 62% of cases, but provided a neuter determiner at more than 25% of the time.

The IC3 items include 15 feminine nouns ending in the feminine suffix, which is phonologically null in the nominative singular and surfaces as −*es* in the plural. Nine of the words in this category have a stem with the thematic vowel /a/, while the rest have the thematic vowel /i/. The adolescents correctly identified these nouns as feminine in an overwhelming 83% of the time, and the majority of their erroneous responses skewed toward the neutral determiner. On the other hand, the child participants were only successful one third of the time, yet their erroneous responses also skewed toward the neuter determiner over half of the time.

From IC4, there are only two learned vocabulary items marked feminine with the null suffix in the nominative singular that manifests as −*is* in the nominative plural. Both stems end in the thematic vowel /i/ and the performance of the two groups followed the same pattern as for IC3.

IC5 is represented with 11 neuter nouns ending in −*o*. The younger group was accurate more than 71% of the time and their erroneous responses were nearly equally distributed between the other two genders (Table 5). The older group was accurate approximately 64% of the time, but their erroneous responses skewed overwhelmingly toward the masculine determiner.

**TABLE 5:** Accuracy and distribution of the children’s answers for the Real Nouns.

	**Children**			
	**%**	**% as**	**% as**	**% as**
	**accurate**	**masculine**	**feminine**	**neuter**
**IC1 −*os* M/F**	26.74	28.1	13.1	58.6
**IC2 −*s* M**	22.57	22.57	15.6	61.8
**IC3 −Ø common F**	33.33	11.39	33.33	55.28
**IC4 −Ø learned F**	14.58	14.58	14.58	70.83
**IC5 −*o* N**	71.21	15.15	13.64	71.21
**IC6 −*i* N**	74.68	14.1	11.22	74.68
**IC7 –*os* N**	70.83	15.63	13.54	70.83
**IC8 −*ma* N**	68.75	8.33	22.92	68.75
**Imparisyllabic −*s* N**	81.25	14.58	4.17	81.25

IC6 includes 13 neuter nouns ending in −*i*. The accuracy of the two groups was comparable but slightly improved to that of IC5, with the exception of the error trend in the older group (adolescents), which this time skewed toward the feminine determiner (Table 6).

**TABLE 6:** Accuracy and distribution of the adolescents’ answers for the Real Nouns.

	**Adolescents**				
	**%**	**% as**	**% as**	**% as**	**% no**
	**accurate**	**masculine**	**feminine**	**neuter**	**answer**
**IC1 −*os* M/F**	48.08	66.6	7.4	26	–
**IC2 −*s* M**	62.82	62.82	9.6	27.6	–
**IC3 −Ø common F**	83.08	2.05	83.08	14.36	0.51
**IC4 −Ø learned F**	88.46	3.58	88.46	7.69	–
**IC5 −*o* N**	64.34	32.17	3.5	64.34	–
**IC6 −*i* N**	68.05	4.73	27.22	68.05	–
**IC7 −*os* N**	42.31	53.85	3.85	42.31	–
**IC8 −*ma* N**	51.28	2.56	46.15	51.28	–
**Imparisyllabic −*s* N**	42.31	50	7.69	42.31	–

IC7 has four neuter nouns ending in −*os*, which follow a distinct inflectional paradigm from masculine or neuter nouns ending in −*os* from IC1. The children continued to perform as they did for IC5 and IC6, correctly providing the neuter determiner; in case of erroneous responses, their mistakes were approximately equally distributed between masculine and feminine. The adolescent participants were accurate well under half of the time and provided a masculine determiner in a majority of the cases (53%).

The six neuter nouns from IC8 are deverbal nouns ending in −*ma*. Here, too, the children mostly provided the correct neuter determiner (69%). Where they replied incorrectly, they favored a feminine (23%) over a masculine determiner (8%). The adolescents provided the correct response at a lower rate (51%). When replying incorrectly, they showed a clear preference for a feminine (46%) over a masculine determiner (3%).

Finally, in the exceptional class of the two imparisyllabic neuter −*s* nouns, the performance of our participants nevertheless does not deviate from what we observed in IC7: The children accurately assigned a neuter determiner in the vast majority of cases (81%) and their errors skewed toward the masculine determiner (15%). In contrast, the adolescents only provided a correct response in a minority of cases (42%) and showed a strong preference for assigning a masculine determiner (50%).

### Accuracy by Prototypicality Class in Real Nouns

The same vocabulary items were placed in the categories proposed by Anastasiadi-Symeonidi and Cheila-Markopoulou (see Table 4). The nouns bearing different nominal suffixes may be prototypical or non-prototypical members of the category that the given nominal suffix defines based on semantic properties, mainly their animacy value and, on some occasions, additionally other semantic factors such as concreteness or diminution. The full word-list is provided as Appendix B. The Tables 7–10 present the aggregate group results. In Appendix C, the Tables C3a–b present individual participant results.

**TABLE 7:** Accuracy, number of items, mean number, standard deviation, and range of correct responses per prototypicality condition in the younger group (children).

	**Children (Accuracy%, *n*, *M*, *SD*, Range)**		
	**Masculine**	**Feminine**	**Neuter**
**+Prototypical,**	45.37, 9, 4.08, 2.06,	47.92, 6, 2.88, 1.57,	70.14, 6, 4.21,
**+Animate**	1–9	0–6	1.28, 2–6
**+Prototypical,**	–	22.5, 5, 1.13, 1.23,	71.3, 18, 12.83,
**−Animate**		0–4	5–18
**−Prototypical,**	–	–	74.31, 6, 4.46, 1.79,
**+Animate**			0–6
**−Prototypical,**	15.74, 9, 1.42, 1.89,	17.36, 12, 2.08, 2.1,	74.31, 6, 4.46,
**−Animate**	0–9	0–7	1.72, 0–6

**TABLE 8:** Accuracy, number of items, mean number, standard deviation, and range of correct responses per prototypicality condition in the older group (adolescents).

	**Adolescents (Accuracy%, *n*, *M*, *SD*, Range)**		
	**Masculine**	**Feminine**	**Neuter**
**+Prototypical,**	78.63, 9, 7.08, 1.8,	89.74, 6, 5.38, 0.77,	64.1, 6, 3.85, 1.63,
**+Animate**	4–9	4–6	1–6
**+Prototypical,**	–	84.62, 5, 4.23, 1.09,	62.82, 18, 11.31,
**−Animate**		2–5	4.82, 3–18
**−Prototypical,**	–	–	64.1, 6, 3.85, 1.95,
**+Animate**			1–6
**−Prototypical,**	60.68 9, 5.46, 3.43,	44.87, 12, 5.38, 2.6,	42.31, 6, 2.54,
**−Animate**	0–9	2–12	1.94, 0–6

**TABLE 9:** Distribution of responses for novel nouns by the younger group (children).

	**Children**		
	**% as**	**% as**	**% as**
	**masculine**	**feminine**	**neuter**
**−*os* ambiguous (M/F/N)**	24.31	16.67	59.03
**−*i* ambiguous (F/N)**	16.03	21.95	62.02
**−*is* M**	20.94	16.23	62.83
**−*as* M**	23.44	15.63	60.94
**−*a* F**	17.15	27.62	55.23
**−*o* N**	18.23	20.31	61.46
**−*ma* N**	19.44	29.86	50.69

**TABLE 10:** Distribution of responses for novel nouns by the older group (adolescents).

	**Adolescents**		
	**% as**	**% as**	**% as**
	**masculine**	**feminine**	**neuter**
**−*os* ambiguous (M/F/N)**	78.71	2.58	18.71
**−*i* ambiguous (F/N)**	7.69	60.9	31.41
**−*is* M**	51.62	14.42	25.96
**−*as* M**	59.62	14.42	25.96
**−*a* F**	4.65	75.19	20.16
**−*o* N**	44.23	4.81	50.96
**−*ma* N**	3.9	46.75	49.35

Prototypical animate nouns may fall into any of the three gender categories. Nine masculine, six feminine, and six neuter nouns are included in this category. Under this classification, the younger group (children) provide correct responses for approximately half of the items in the masculine and feminine conditions but had an accuracy of slightly over 70% in the neuter condition. The older group (adolescents) was very accurate in the feminine condition, approaching 90%, and also in the masculine condition with over 78% of correct responses. Their accuracy in the neuter condition was well above chance but not as remarkable as in the previous two.

The prototypical inanimate class does not include any masculine nouns but consists of five feminine and 18 neuter nouns. In this category, the younger participants performed worse in the feminine condition, with correct responses making up only slightly over one fifth of the total, while their accuracy in the neuter condition was similar to their accuracy in the prototypical animate category. The older participants’ accuracy was not remarkably different compared to the previous category.

The non-prototypical animate category includes only six neuter nouns and the two groups showed comparable accuracy as in the neuter conditions in both prototypical categories.

Finally, the non-prototypical inanimate category includes nouns from all three genders, of which nine were masculine, 12 were feminine, and six were neuter. The nouns in this category elicited some of the lowest accuracy rates in the prototypicality analysis. The younger participants provided accurate responses well below one fifth of the time in the masculine and feminine conditions, but they retained their previous levels of accuracy in the neuter condition. The older participants, who previously were remarkably accurate in the feminine and masculine conditions, did not perform as accurately here, correctly responding approximately 45 and 61% of the time, in the respective categories. Their relatively lower accuracy when it comes to neuter nouns was more pronounced in this category, reaching only slightly over 42%.

When looking at the performance of individual participants, we observe higher deviations from the group accuracy rate, with extremes such as a standard deviation of 12.83 in the Prototypical Inanimate Neuter category for child participants. Overall, under the prototypicality analysis, no age group appears to consistently achieve more heterogenous results.

### Accuracy by Nominal Suffix in Novel Nouns

The novel nouns, by definition devoid of semantic associations, were only analyzed in terms of morphological form. Additionally, because the participants did not know these words beforehand, homophonous suffixes that may belong to separate inflectional classes–namely −*os* (M/F vs. N) and −*i* (F vs. N)–were aggregated together, since the participants could not be aided by familiarity with inflected forms.

The −*os* category, which consisted of twelve items, was expected to elicit all three gender-marked determiners. Nevertheless, both groups showed a clear preference, albeit a different one. In nearly 60% of the cases, children preferred assigning the neuter gender to these words. The adolescents, in contrast, preferred the masculine determiner in nearly 80% of the cases. In both cases, the feminine determiner was the least popular response, although the distance between that and the other two options was more striking in the adolescents’ responses.

The −*i* category included 12 items that were expected to elicit either feminine or neuter determiners. The two groups indeed preferred the two options in most cases, with masculine determiners making up only 16% of the responses in the younger group, and almost 8% in the older group. The two age groups once again showed distinct preferences, with the children preferring the neuter option in slightly over 62% of the cases, and the adolescents the feminine in slightly under 61%.

The −*is* category consists of eight nouns that were expected to elicit only masculine determiners. The expectation was subverted by both groups, as only approximately 21% of the items elicited a masculine determiner in the younger group, and the older group provided one only slightly above 51% of the time. The distribution of erroneous responses favored once more the neuter determiner, especially in the younger group, while the older participants also provided a non-trivial amount of feminine determiners in this category.

The −*as* category has eight items, all expected to elicit masculine determiners. Both groups performed comparably to the −*is* category, with the older group correctly producing a masculine determiner in 8% more of the cases. The younger group only saw a 2.5% improvement, but still overwhelmingly preferred the erroneous neuter determiner.

The −*a* category consists of 10 items expected to elicit feminine determiners. The older participants successfully provided them in three fourths of the cases, with most of their erroneous replies favoring the neuter determiner. The younger participants provided the correct determiner in slightly under 28% of the cases, replying mostly with the neuter determiner in over 55% as well as with a non-trivial amount of masculine determiners.

The −*o* category has eight test items expected to elicit a neuter determiner. Neither group performed clearly as expected. The children produced the neuter determiner approximately 60% as it did in the previous categories, while the adolescents were almost equally split between neuter and masculine.

The −*ma* category included six items expected to elicit a neuter determiner. The two groups also defied this expectation, with the younger group accurately responding only 51% of the time, and their erroneous responses skewing toward the feminine determiner, while the older group was once more nearly equally split, this time between feminine and neuter.

## Discussion

By employing a task that directly elicits a gender-marked determiner, we were able to detect a difference in performance both between two age groups of heritage speakers, as well as between heritage speakers and homeland speakers where comparisons are possible.

The results of this study highlight a likely contribution of language exposure (by proxy of age) and of specifically phonological–rather than expected broadly morphological–cues in the process of gender assignment in both the real-word and the novel-word tasks. In turn, the expected facilitatory effects of semantic information as introduced by the concept of prototypical gender values were not as pronounced in the real-word task.

With regard to the contribution of morphological information, across the categorizations of both the real vocabulary items and the novel nouns, the children tended to respond with the neuter determiner. The distribution of their responses suggests that the retreat to neuter is not indicative of preserved knowledge in favor of neuter vocabulary items but rather of a retreat to a default due to uncertainty regarding the gender value that the form encodes. This finding is similar to what Alexiadou et al. (2021) observed for adolescent and adult heritage speakers of Greek in the United States on the basis of a narration task. But unlike what has been proposed in that paper, in our study, the effect cannot be attributed to difficulties with the establishment of agreement chains.

The facilitatory effect of prototypicality in this group was restricted to prototypical masculine and feminine items, but it was not strong enough to enable them to perform clearly above chance level. Animacy showed an additional facilitatory effect for the feminine items, although the absence of non-prototypical animate items in this study does not allow us to tease apart the influence of the two factors. Prototypicality effects were completely absent in the cause of neuter items, where correct responses were uniformly high. The retreat to a default supports the Tsimpli and Hulk’s (2013) assessment that neuter is the learner default in Greek.

The adolescents appear to be very sensitive to phonological cues in both the real and the novel noun conditions, at the expense of expected morphological cues that are used to define the various ICs. In the real noun condition, we observe a strong tendency to interpret all /s/-ending suffixes as masculine, and secondarily as neuter, which leads to their high accuracy rate for masculine nouns; but it impedes their accuracy in the rarer feminine nouns ending in −*os*. They also appear less sensitive to the paradigm difference between M + F −*os* vs. N −*os*, which may indicate less reliable access to the inflected forms of the nouns.

At the same time, the adolescents also appear to strongly associate nominal forms ending in the vowels /i/ and /a/ with feminine gender, which manifests as remarkable accuracy in the feminine noun conditions; but it also impedes their accuracy when it comes to deverbal nouns ending in −*ma*. We hypothesize that this is due to a reanalysis of −*ma* as a stem with a thematic vowel /a/ and the feminine null nominal suffix, contrary to Varlokosta’s (2011) categorization of the two as unambiguous. The sensitivity to prototypicality is strongly pronounced between prototypical and non-prototypical inanimate feminine nouns, where non-prototypical feminine nouns are correctly identified as feminine at below or slightly above chance level, while prototypical feminine nouns were consistently identified correctly, more so than any other category in this analysis. A smaller animacy effect could also be observed for non-prototypical neuter items.

More broadly, we note that neither the paradigm-focused categorization system from Ralli (1994) nor the semantic-morphological system from Anastasiadi-Symeonidi and Cheila-Markopoulou (2003) allow us to fully capture the performance patterns of the heritage speakers. With regard to Ralli’s system, we have discussed an apparent reliance of the heritage speakers on the phonological endings of the test items, giving rise to ambiguities that are not expected in the production of their homeland counterparts. As this categorization system relies on access to the full paradigm, we consider unavailability or degraded access to the full paradigm to be a possible explanation for the lower accuracy of even the older heritage speakers compared to their homeland counterparts. We believe that similar elicitation tasks that attempt to elicit different combinations of gender, case, and number values can provide the data needed to pursue this line of investigation. With regard to Anastasiadi-Symeonidi & Cheila-Markopoulou’s system, we have discussed some tentative indications of increased accuracy for prototypical nouns of their respective genders. We also note that under this categorization system, the ambiguities between nouns of different inflectional classes with homophonous endings are not surprising as the system does not account for inflectional paradigm differences. We remain reserved about the significance of those observations, since the test items were not balanced across the two dimensions of prototypical and animacy.

Finally, we acknowledge again a number of limitations that arise from the preliminary nature of our study and the constraints inherent to the study of heritage populations. The small number of participants and the heterogeneity the sample exhibits did not allow us to robustly examine the observations discussed above and establish their statistical significance. However, our findings in the child group do align with other studies of learner Greek, while those in the adolescent group point to differences from the homeland group. This latter pattern is quite robust as shown in section “Results.”

## Conclusion

Our study sought, on the one hand, to establish whether a determiner elicitation task would be an appropriate tool for investigating the principles guiding grammatical gender assignment in a heritage language population. On the other hand, it aimed to provide data regarding the accuracy of this group of Heritage Greek speakers with English as their dominant language, and compare their performance to that of end-state (monolingual Greek) homeland speakers. The task does appear to be appropriate: The relatively effortless and early acquisition of the grammatical gender feature in homeland speakers is not replicated in a heritage language environment, although an increased accuracy of the adolescent group can be observed in some of the inflectional categories.

By examining the error types in the data in both the real and the novel word sub-tasks, we hoped to identify a difference in the degree of reliance upon distinct grammatical gender assignment mechanisms. In the child group, the retreat to the default neuter dominates and other gender cues have limited influence. The response patterns of the adolescent group suggest that among the possible sources of a gender value, a greater sensitivity was shown to the purely phonological properties of the inflectional suffix, giving rise to unexpected ambiguities that are not encountered in (monolingual) Greek homeland performance patterns, such as that of neuter −*ma* and feminine −*a* that would be eliminated on morphological grounds.

Finally, by utilizing Anastasiadi-Symeonidi and Cheila-Markopoulou’s (2003) categorization principles based on prototypicality in our alternative analysis, we attempted to evaluate the degree in which purely semantic principles could also be at play. We could identify a strong sensitivity to a prototypicality effect only in the case of feminine vocabulary items, while a weaker sensitivity to animacy was seen in non-prototypically neuter items.

The preliminary results of this study have highlighted the relevance of access to the inflectional paradigms (case and number) of the nominal suffixes in question. This holds particularly for the reliance on strictly phonological cues by the heritage speaker population in such a way that gives rise to ambiguous gender-marking situations not found in (monolingual Greek) homeland speakers’ productions. We suggest that further research in the gender assignment patterns of Heritage Greek speakers which is informed by models of organization of the mental lexicon with regard to inflectional paradigms.

## Data Availability Statement

The raw data supporting the conclusions of this article will be made available by the authors, without undue reservation.

## Ethics Statement

The studies involving human participants were reviewed and approved by Cyprus National Bioethics Committee. Written informed consent to participate in this study was provided by the participants’ legal guardian/next of kin.

## Author Contributions

All authors listed have made a substantial, direct and intellectual contribution to the work, and approved it for publication.

## Conflict of Interest

The authors declare that the research was conducted in the absence of any commercial or financial relationships that could be construed as a potential conflict of interest.

## Publisher’s Note

All claims expressed in this article are solely those of the authors and do not necessarily represent those of their affiliated organizations, or those of the publisher, the editors and the reviewers. Any product that may be evaluated in this article, or claim that may be made by its manufacturer, is not guaranteed or endorsed by the publisher.
