# Supplementary material for: Assignment of Grammatical Gender in Heritage Greek
Source: Front Psychol. 2021 Oct 8;12:717449. doi: 10.3389/fpsyg.2021.717449 (PMC8531586; doi:10.3389/fpsyg.2021.717449)
Supplement: Supplementary file 1 [file Data_Sheet_1.pdf]

Appendix A: Real and Novel Nouns by IC or Suffix

The words are listed in their original Greek spelling, followed by their phonemic transcription, and a conventional English translation for the real nouns.

IC1 (–os M/F)
άνθρωπος - ánθropos - human
έξοδος - éksoðos - exit
αδελφός - aðelfós - brother
δρόμος - ðrómos - road
είσοδος - ísoðos - entry
άμμος - ámmos - sand
θείος - θíos - uncle
πρόοδος - próoðos - progress
άνεμος - ánemos - wind
οδός - oðós - street
ήπειρος - ípiros - continent
ουρανός - uranós - sky

IC2 (–s M)
χορευτής - xoreftís – dancer
καθρέφτης - kaθréftis - mirror
μαθητής - maθitís - student
νιπτήρας - niptíras - washbasin
ελέφαντας - eléfantas - elephant
νεροχύτης - neroçítis - sink
ιππότης - ippótis - knight
χάρτης - xártis - map
καστανάς - kastanás - chestnut vendor
κουμπαράς - kumbarás - piggy bank
μουσακάς - musakás - moussaka (dish)
πατέρας - patéras - father

IC3 (–Ø F common)
αγελάδα - ajeláða - cow
βρύση - vrísi - faucet
μητέρα - mitéra - mother
μπάλα - bála - ball
κόρη - kóri - daughter
δασκάλα - ðaskála - teacher
νύφη - nífi - bride
δόξα - ðóksa - glory
ομορφιά - omorfjá - beauty
αμμουδιά - ammuðjá - beach
μαγειρική - majirikí - cookery
πατάτα - patáta - potato
καρέκλα - karékla - chair
ζώνη - zóni - belt
ζάχαρη - záxari - sugar

IC4 (–Ø F learned)
τίγρη - tíɣri - tiger
πόλη - póli - city

IC5 (–o N)
δωμάτιο - ðomátio - room
βουνό - vunó - mountain
μήλο - mílo - apple
πρόβατο - próvato - sheep
ζώο - zóo - animal
θρανίο - θranío - desk
δώρο - ðóro - gift
βασιλόπουλο - vasilópulo - young prince
παράθυρο - paráθiro - window
μωρό - móro - baby
γειτονόπουλο - jitonópulo - young neighbor

IC6 (–i N)
σταφύλι - stafíli - grape
τραπέζι - trapézi - table
δόντι - ðónti - tooth
πόδι - póði - foot
πουλί - púli - bird
χταπόδι - xtapóði - octapus
κουτί - kutí - box
μπαλόνι - balóni - baloon
αγόρι - aɣóri - boy
γουρούνι - ɣurúni - pig
χελιδόνι - çeliðóni - swallow
αρνί - arní - lamb
παιδί - peðí - child

IC7 (–os N)
λάθος - láθos - mistake
δάσος - ðásos - forest
ξίφος - ksífos - sword
βάρος - város - weight

IC8 (–ma N)
κύμα - císma - wave
μάθημα - máθima - lesson
γράμμα - ɣrámma - letter
πάτωμα - pátoma - floor
αίμα - éma - blood
χρώμα - xróma - color

Imper. Neut –s
κρέας - kréas - meat
φως - fós - light

Ambiguous –*os*
ταγερός - tajerós
ολενός - olenós
ρεβός - révos
άφουδος - afuðós
τουκνός - tuknós
βέδος - véðos
ψέθος - pséθos
πεγατός - peɣátos
γέτρουχος - jetrúxos
γλένος - ɣlénos
διγανός - ðiɣanós
ζουρός - zurós

Ambiguous –*i*
τάρη - tári
βιακοπή - viakopí
βουντί - vundí
πεκί - pecí
ναφική - nafićí
ντολάνι - doláni
δράνη - ðráni
ταρί - tarí
νεβαζί - nevázi
κεραλίνη - ceralíni
τουμάλι - tumáli
νέπη - népi

Masc –*is*
νεβάτης - nevátis
χεπέτης - çepétis
πεφάτης - pefátis
λόχτης- lóxtis
μαλουφύτης - malufítis
πλετής - pletís
γέρτης - jértis
ρεχτής - rextís

Masc –*as*
κουπέρας - kupéras
άλωας - áloas
ζαρήνας - zirínas
πεσκονάς - peskonás
μαδεκάς - maðekás
πλέας - pléas
γέχας - jexás
θέροκας - θérokas

Fem –*a*
νεπέρα - nepéra
αβιλέδα - aviléða
δέθερα - ðeθéra
δράσσα - ðrássa
γκάρα - gára
αροχιά - aroçá
γοργιά - ɣorjá
τεργιά - terjá
πιγκελιά - piɟeljá
κλοτέζα - klotéza

Neut –*o*
ζονό - zonó
φρεμίο - fremío
γεγρίο - jeɣrío
κεράφυρο - keráfiro
κεύκο - kéfko
τάκελο - tácelo
φάργιο - fárjo
γάνδρο - ɣánðro

Neut –*ma*
πέφισμα - péfisma
νέδημα - néðima
τέκωμα - tékoma
τούμα - túma
γλώμα - ɣlóma
φλάμμα - flámma
