# Supplementary material for: Assignment of Grammatical Gender in Heritage Greek
Source: Front Psychol. 2021 Oct 8;12:717449. doi: 10.3389/fpsyg.2021.717449 (PMC8531586; doi:10.3389/fpsyg.2021.717449)
Supplement: Supplementary file 2 [file Data_Sheet_2.PDF]

Appendix B: Real Nouns by Prototypicality

+Prototypical

+Animate

Masc *–s*

άνθρωπος - ánθropos - human
χορευτής χορευτής - xoreftís - dancer
πατέρας - patéras - father
αδελφός - aðelfós - brother
μαθητής - maθitís - student
θείος - θíos - uncle
ελέφαντας - eléfantas - elephant
ιππότης - ippótis - knight
καστανάς - kastanás - chestnut vendor

Fem *–a/i/u*

αγελάδα - ajeláða - cow
μητέρα - mitéra - mother
κόρη - kóri - daughter
δασκάλα - ðaskála - teacher
νύφη - nífi - bride
τίγρη - tíɣri - tiger

Neut *–o/i/a*

βασιλόπουλο - vasilópulo - young prince
μωρό - móro - baby
γειτονόπουλο - jitonópulo - young neighbor
αγόρι - aɣóri - boy
αρνί - arní - lamb
παιδί - peðí - child

–Animate

Fem *–a/i/u*

δόξα - ðóksa - glory
ομορφιά - omorfjá - beauty
αμμουδιά - ammuðjá - beach
μαγειρική - majirikí - cookery
πόλη - póli - city

Neut *–o/i/a*

δωμάτιο - ðomátio - room
σταφύλι - stafíli - grape
βουνό - vunó - mountain
δόντι - ðónti - tooth
τραπέζι - trapézi - table
κύμα - címa - wave
μήλο - mílo - apple
πόδι - póði - foot
μάθημα - máθima - lesson
θρανίο - θranío - desk
δώρο - ðóro - gift
γράμμα - ɣrámma - letter
πάτωμα - pátoma - floor

αίμα - éma - blood
παράθυρο - paráθiro - window
κουτί - kutí - box
μπαλόνι - balóni - baloon
χρώμα - xróma - color

Neut –*o/i*
πρόβατο - próvato - sheep
ζώο - zóo - animal
+Animate πουλί - púli - bird
χταπόδι - xtapóði - octapus
γουρούνι - ɣurúni - pig
χελιδόνι - çeliðóni - swallow

Masc –*s*
μουσακάς - musakás - moussaka (dish)
καθρέφτης - kaθréftis - mirror
δρόμος - ðrómos - road
ουρανός - uranós - sky
νιπτήρας - niptíras - washbasin
νεροχύτης - neroçítis - sink
άνεμος - ánemos - wind
χάρτης - xártis - map
κουμπαράς - kumbarás - piggy bank

–Prototypical **Fem –*a/i/u/s***
βρύση - vrísi - faucet
μπάλα - bála - ball
πατάτα - patáta - potato
–Animate καρέκλα - karékla - chair
ζώνη - zóni - belt
ζάχαρη - záxari - sugar
έξοδος - éksoðos - exit
είσοδος - ísoðos - entry
άμμος - ámmos - sand
πρόοδος - próoðos - progress
οδός - oðós - street
ήπειρος - ípiros - continent

Neut –*s*
λάθος - láθos - mistake
δάσος - ðásos - forest
ξίφος - ksífos - sword
βάρος - város - weight
κρέας - kréas - meat
φως - fós - light
