# Supplementary material for: Assignment of Grammatical Gender in Heritage Greek
Source: Front Psychol. 2021 Oct 8;12:717449. doi: 10.3389/fpsyg.2021.717449 (PMC8531586; doi:10.3389/fpsyg.2021.717449)
Supplement: Supplementary file 3 [file Data_Sheet_3.PDF]

Appendix C: Accuracy Per Participant

Table C1a: Accuracy of child participants per IC (real nouns)

	P1	P2	P3	P4	P5	P6	P7	P8	P9	P10	P11	P12	P13	P14	P15	P16	P17	P18	P19	P20	P21	P22	P23	P24
**IC1 –*os* M/F (12)**	0.33	0.33	0.25	0.25	0.25	0.5	0.42	0.25	0.17	0.33	0.08	0.25	0.5	0.25	0.17	0.17	0.42	0.17	0.33	0.08	0.17	0.25	0.5	0
**IC2 –*s* M (12)**	0.17	0.17	0.17	0.17	0.17	1	0.67	0.17	0.25	0.25	0.25	0.33	0.08	0.08	0.08	0.08	0.25	0	0.25	0.17	0.08	0.17	0.17	0.25
**IC3 –Ø F (15)**	0.13	0.4	0.27	0.33	0.2	0.73	0.8	0.2	0.33	0.27	0.27	0.2	0.33	0.4	0.27	0.27	0.53	0.13	0.27	0.2	0.2	0.33	0.47	0.47
**IC4 –Ø F (2)**	0	0.5	0	0	0	0	1	0	0	0.5	0.5	0	0	0	0	0	0.5	0	0	0	0	0	0.5	0
**IC5 –*o* N (11)**	0.73	0.55	0.64	0.82	1	0.73	0.45	0.82	1	0.82	0.73	0.36	0.55	0.82	1	1	0.27	1	0.36	0.73	0.91	0.91	0.36	0.55
**IC6 –*i* N (13)**	0.85	0.54	0.77	0.92	0.92	0.85	0.85	1	0.85	0.62	0.46	0.38	0.54	0.92	0.92	0.92	0.38	0.92	0.69	0.54	0.92	1	0.54	0.62
**IC7 –*os* N (4)**	0.75	1	0.75	1	1	0	0.5	0.75	0.75	1	0	0.75	0.75	1	1	1	0.5	1	0.25	0.5	1	1	0.25	0.5
**IC8 –*ma* N (6)**	1	0.83	1	0.67	1	0	0.83	0.83	1	0.5	0.5	0.67	0.5	1	0.5	0.5	0.83	0.83	0.83	0	1	1	0.33	0.33
**Imparisyllabic –*s* N (2)**	1	0.5	1	1	1	0	1	1	1	0.5	0.5	1	0.5	1	1	1	1	1	0.5	0.5	1	1	0.5	1
**Overall accuracy**	***0.55***	***0.54***	***0.54***	***0.57***	***0.62***	***0.42***	***0.72***	***0.56***	***0.59***	***0.53***	***0.37***	***0.44***	***0.42***	***0.61***	***0.55***	***0.55***	***0.52***	***0.56***	***0.39***	***0.3***	***0.59***	***0.63***	***0.4***	***0.41***

Table C1b: Accuracy of adolescent participants per IC (real nouns)

	P1	P2	P3	P4	P5	P6	P7	P8	P9	P10	P11	P12	P13
**IC1 –*os* M/F (12)**	0.58	0.5	0.42	0.42	0.5	0.33	0.25	1	0.5	0.42	0.25	0.5	0.58
**IC2 –*s* M (12)**	0.25	1	0.42	0.42	0.83	0.33	0.17	1	1	0.58	0.25	1	0.92
**IC3 –Ø F (15)**	0.8	1	1	0.87	1	0.67	0.6	1	0.93	0.8	1	0.4	0.73
**IC4 –Ø F (2)**	0.5	1	1	1	1	1	0.5	1	1	0.5	1	1	1
**IC5 –*o* N (11)**	0.45	1	0.55	0.55	1	0.09	0.36	1	0.82	0.27	0.73	1	0.55
**IC6 –*i* N (13)**	0.62	1	0.62	0.46	0.15	0.69	0.54	1	1	0	1	0.92	0.85
**IC7 –*os* N (4)**	0.25	0	0.5	0.5	0.25	0.25	0.75	0.75	0.5	0.5	1	0	0.25
**IC8 –*ma* N (6)**	0.67	1	0	0.17	0	0.17	0.83	1	1	0.33	0	0.5	1
**Imparisyllabic –*s* N (2)**	0	0	0.5	1	0	0.5	0.5	1	1	0	1	0	0
**Overall accuracy**	***0.46***	***0.72***	***0.55***	***0.6***	***0.53***	***0.45***	***0.5***	***0.97***	***0.86***	***0.38***	***0.69***	***0.59***	***0.65***

Table C2a: Accuracy of child participants per phonological ending (novel nouns)

	P1	P2	P3	P4	P5	P6	P7	P8	P9	P10	P11	P12	P13	P14	P15	P16	P17	P18	P19	P20	P21	P22	P23	P24
**-os**	1	1	1	1	1	1	1	1	1	1	1	1	1	1	1	1	1	1	1	1	1	1	1	1
**-i**	0.58	0.75	0.5	0.75	0.83	0.83	1	0.92	1	0.75	0.75	0.67	0.92	0.83	1	1	0.75	1	0.92	0.83	1	0.92	0.58	1
**-is**	0.13	0.63	0	0.25	0.25	1	0.5	0	0	0.5	0.25	0.25	0.13	0.38	0	0	0.25	0	0.13	0	0	0.25	0	0.13
**-as**	0.13	0.5	0.5	0.38	0.25	1	0.38	0	0	0.25	0.25	0.38	0.13	0.38	0	0	0.25	0	0.25	0.25	0	0	0.38	0
**-a**	0.2	0	0.4	0.2	0	1	0.6	0	0	0.1	0.5	0.2	0.3	0.4	0.7	0.7	0.4	0	0.3	0.3	0	0	0.2	0.1
**-o**	0.25	0.63	0.13	0.63	0.5	0.25	0.63	1	1	0.13	0.75	0.75	0.38	0.63	1	1	0.13	1	0.25	0.75	1	0.5	0.63	0.88
**-ma**	0.17	0.5	0.17	0.83	1	0	0.67	0.83	1	0.5	0.33	0.67	0.33	0.33	0.17	0.17	0.33	1	0.17	0.33	1	0.33	0.33	1
**Overall accuracy**	***0.35***	***0.57***	***0.38***	***0.58***	***0.55***	***0.73***	***0.68***	***0.54***	***0.57***	***0.46***	***0.55***	***0.56***	***0.45***	***0.56***	***0.55***	***0.55***	***0.44***	***0.57***	***0.43***	***0.5***	***0.57***	***0.43***	***0.45***	***0.59***

Table C2b: Accuracy of adolescent participants per phonological ending (novel nouns)

	P1	P2	P3	P4	P5	P6	P7	P8	P9	P10	P11	P12	P13
**-os**	1	1	1	1	1	1	1	1	1	1	1	1	0.92
**-i**	0.75	1	0.83	0.92	1	0.92	1	1	1	0.92	0.75	0.92	1
**-is**	0.25	1	0.5	0.25	0.88	0.25	0.25	0.75	0.75	0	0.25	0.63	1
**-as**	0.25	1	0.25	0.25	1	0.38	0.13	1	0.88	0.63	0	1	1
**-a**	0.7	1	1	0.9	0.8	0.9	0.3	1	0.9	0.5	1	0.3	0.4
**-o**	0.5	1	0.25	0.13	0.88	0.38	0.38	1	0.88	0.13	0	1	0.13
**-ma**	0.33	1	0.33	0.33	0.17	0.17	0.5	0.83	0.5	0.83	0	0.83	0.5
**Overall accuracy**	***0.54***	***1***	***0.6***	***0.54***	***0.82***	***0.57***	***0.51***	***0.94***	***0.84***	***0.57***	***0.43***	***0.81***	***0.71***

Table C3a: Accuracy of child participants per prototypicality condition[1] (real nouns)

	P1	P2	P3	P4	P5	P6	P7	P8	P9	P10	P11	P12	P13	P14	P15	P16	P17	P18	P19	P20	P21	P22	P23	P24
**+Pro+An_M**	0.33	0.33	0.67	0.44	0.44	1	1	0.56	0.33	0.56	0.11	0.33	0.33	0.56	0.44	0.44	0.67	0.33	0.33	0.22	0.44	0.67	0.22	0.11
**+Pro+An_F**	0.17	0.33	0.67	0.67	0.33	0.5	1	0.5	0.5	0.5	0.5	0	0.17	0.67	0.5	0.5	1	0.33	0.33	0	0.5	0.83	0.67	0.33
**+Pro+An_N**	1	0.5	0.67	0.83	0.83	0.67	0.33	0.83	0.83	0.33	0.67	1	0.83	1	0.83	0.83	0.5	0.83	0.33	0.67	0.67	0.83	0.67	0.33
**+Pro-An_F**	0	0.6	0	0.2	0.2	0.8	0.8	0	0.4	0.2	0.2	0.2	0.2	0	0.2	0.2	0	0	0.2	0.2	0	0	0.2	0.6
**+Pro-An_N**	0.83	0.72	1	0.72	1	0.44	0.89	0.89	0.94	0.67	0.56	0.39	0.33	0.89	0.78	0.78	0.44	0.94	0.72	0.28	1	1	0.33	0.56
**-Pro+An_N**	0.83	0.33	0.17	1	1	1	0.67	1	1	0.83	0.67	0	0.67	0.83	1	1	0.33	1	0.5	0.83	1	1	0.67	0.5
**-Pro-An_M**	0.22	0.11	0	0.22	0.11	1	0.33	0.11	0.11	0.11	0.22	0.11	0.33	0	0	0	0.22	0	0.11	0	0	0	0.22	0.22
**-Pro-An_F**	0.25	0.33	0	0	0	0.33	0.42	0	0	0.17	0.17	0.33	0.42	0.17	0	0	0.33	0	0.25	0.25	0	0	0.58	0.17
**-Pro-An_N**	0.83	0.83	0.83	1	1	0	0.67	0.83	0.83	0.83	0.17	0.83	0.67	1	1	1	0.67	1	0.33	0.5	1	1	0.33	0.67
**Overall accuracy**	***0.5***	***0.46***	***0.44***	***0.57***	***0.55***	***0.64***	***0.68***	***0.52***	***0.55***	***0.47***	***0.36***	***0.36***	***0.44***	***0.57***	***0.53***	***0.53***	***0.46***	***0.49***	***0.35***	***0.33***	***0.51***	***0.59***	***0.43***	***0.39***

Table C3a: Accuracy of adolescent participants per prototypicality condition (real nouns)

	P1	P2	P3	P4	P5	P6	P7	P8	P9	P10	P11	P12	P13
**+Pro+An_M**	0.56	1	0.67	0.67	0.89	0.67	0.56	1	1	0.89	0.44	1	0.89
**+Pro+An_F**	0.67	1	1	1	1	0.83	0.83	1	1	0.83	1	0.67	0.83
**+Pro+An_N**	0.67	1	0.5	0.5	0.67	0.33	0.33	1	0.67	0.17	0.67	1	0.83
**+Pro-An_F**	0.8	1	1	1	1	0.6	0.6	1	1	0.6	1	0.4	1
**+Pro-An_N**	0.61	1	0.44	0.5	0.33	0.39	0.56	1	1	0.17	0.67	0.78	0.72
**-Pro+An_N**	0.33	1	0.5	0.17	0.5	0.33	0.67	1	1	0.17	0.83	1	0.83
**-Pro-An_M**	0.22	1	0.44	0.44	0.89	0.22	0	1	1	0.44	0.22	1	1
**-Pro-An_F**	0.67	0.5	0.5	0.33	0.5	0.33	0.17	1	0.42	0.42	0.5	0.17	0.33
**-Pro-An_N**	0.17	0	0.5	0.67	0.17	0.33	0.67	0.83	0.67	0.33	1	0	0.17
**Overall accuracy**	***0.52***	***0.83***	***0.62***	***0.59***	***0.66***	***0.45***	***0.49***	***0.98***	***0.86***	***0.45***	***0.7***	***0.67***	***0.73***

1 ± Prototypical (Pro); ±Animate (An); Masculine (M), Feminine (F), Neuter (N)
